# Advances in *Legionella* Control by a New Formulation of Hydrogen Peroxide and Silver Salts in a Hospital Hot Water Network

**DOI:** 10.3390/pathogens8040209

**Published:** 2019-10-29

**Authors:** Luna Girolamini, Ada Dormi, Tiziana Pellati, Paolo Somaroli, Davide Montanari, Andrea Costa, Francesca Savelli, Andrea Martelli, Antonella Grottola, Giulia Fregni Serpini, Sandra Cristino

**Affiliations:** 1 Department of Biological, Geological, and Environmental Sciences, BiGeA, University of Bologna, 40126 Bologna, Italy; luna.girolamini2@unibo.it; 2 Department of Medical and Surgical Science, DIMEC, University of Bologna, 40126 Bologna, Italy; ada.dormi@unibo.it; 3 GVM Care & Research, Lugo di Ravenna, 48022 Ravenna, Italy; tpellati@gvm-engineering.it (T.P.); psomaroli@gvm-engineering.it (P.S.); 4 Eta-Beta S.r.l., 47122 Forlì, Forlì Cesena, Italy; manutenzione-mch@gvmnet.it (D.M.); costa.andrea@etabetasrl.com (A.C.); 5 Water Team S.r.l., 47522 Cesena, Forlì Cesena, Italy; francesca@waterteam.it (F.S.); andream@waterteam.it (A.M.); 6 Regional Reference Laboratory for Clinical Diagnosis of Legionellosis, Unit of Microbiology and Virology, Modena University Hospital, 41125 Modena, Italy; grottola.antonella@aou.mo.it (A.G.); fregniserpini.giulia@aou.mo.it (G.F.S.)

**Keywords:** WTP 828, *Legionella*, risk assessment plan, water quality, microbial analysis, chemical analysis

## Abstract

*Legionella* surveillance is an important issue in public health, linked to the severity of disease and the difficulty associated with eradicating this bacterium from the water environment. Different treatments are suggested to reduce *Legionella* risk, however long-term studies of their efficiency are lacking. This study focused on the activity of a new formulation of hydrogen peroxide and silver salts, WTP828, in the hospital hot water network (HWN) to contain *Legionella* contamination during two years of treatment. The effectiveness of WTP828 was tested measuring physical-chemical and microbiological parameters such as *Legionella*, *Pseudomonas aeruginosa* (*P. aeruginosa*), and a heterotopic plate count (HPC) at 36 °C. *Legionella* isolates were identified by serotyping and genotyping. WTP 828 induced a reduction in *Legionella*–positive sites (60% to 36%) and contamination levels (2.12 to 1.7 log_10_ CFU/L), with isolates belonging to *L. pneumophila* SG1 (ST1 and ST104), *L. anisa* and *L. rubrilucens* widely distributed in HWN. No relevant contamination was found for other parameters tested. The long-term effect of WTP828 on *Legionella* containment suggest the easy and safe application of this disinfectant, that combined with knowledge of building characteristics, an adequate environmental monitoring and risk assessment plan, become the key elements in preventing *Legionella* contamination and exposure.

## 1. Introduction

Hot and cold water systems (e.g., tap water installations, distribution systems, and cooling towers) are important sources of nosocomial and community-acquired infections caused by opportunistic waterborne pathogens. Among them, *Legionella* spp. are water-based organisms that cause lung infections when inhaled in an aerosol form [1].

Several national standards have been established to ensure a high water quality using disinfection techniques that control and prevent the colonization of water systems by *Legionella* [2]. A wide variety of disinfection techniques, including chemical disinfection, ultraviolet (UV) light, and high temperature, have been employed worldwide to reduce the risk of legionellosis [3,4].

In Italy, Legionnaires’ disease (LD) is a class II statutorily notifiable disease [5]; since 1983, it has also been subject to a reporting system designed to collect detailed information about contamination cases, which is held in a national register at the Istituto Superiore di Sanità (ISS), Italy. However, according to ISS annual reports, the number of LD cases is under-diagnosed and under-reported, leading to a significant underestimation of the real incidence of LD. In 2017, the incidence rate was 33.2 cases per million persons [6].

Following publication of the new Italian Guidelines for the Control and Prevention of legionellosis in May 2015 [7], the importance of a surveillance program encompassing all facilities at risk of LD (hospitals, healthcare facilities, dental units, hotels, tourist facilities, and spas) has been acknowledged, and this program has been implemented. The guidelines support the development of a risk assessment plan based on an evaluation of “risk” and also emphasize the need for an adequate environmental surveillance plan that includes an appropriate number of sites that are potential sources of *Legionella*.

A recent multicenter study performed by Montagna et al. [8] has demonstrated, as the main methods to perform *Legionella* prevention and control for the water network, were shock treatment and chlorination.

The shock treatment consists of a thermal disinfection of hot-water distribution systems performed at a temperature between 70–80 °C starting from the hot water storage heater. The temperature must be maintained in all outlets, faucets, and shower heads at least 30 min at 60–65 °C, for three consecutive days [7,9,10].

Several studies showed as the main disadvantage of shock treatment is its transitorily effect on bacterial community structure, e.g., biofilm, that was not removed preserving pathogenic *Legionella* niche [11,12,13].

Chlorine is the most common chemical disinfectant used in water (including drinking water), acts as an oxidizing agent, and reacts with several cellular constituents including the cell membrane of microbes. To perform *Legionella* control, plumbing water systems can be treated using chlorine as a shock hyperchlorination (residual chlorine concentration at distal outlets of 20–50 mg/L) or as continuous treatment using a concentration of 1–2 mg/L [10]. Although different studies have shown good performance using these methods to assess *Legionella* contamination, a reduction of effectiveness over a long-term period was consistently demonstrated [10,14,15,16]. However, increasing evidence suggests that humans are exposed to residual byproducts of water chlorination such as disinfection byproducts (DBPs) through drinking-water, oral, dermal, and inhalational contact. During the chlorination, especially by hypochlorous acid and hypobromous acid, the reaction with naturally occurring organic matter present in raw water supplies, create many water DBPs, including the four primary trihalomethanes: chloroform (CHCl_3_), bromodichloromethane (CHCl_2_Br), dibromochloromethane (CHClBr_2_), and bromoform (CHBr_3_), that can have adverse effects on human health [17,18,19,20].

Disinfection methods other than chlorination have been suggested for *Legionella* control in water, such as ozone treatment, copper and silver (Ag^+^) ionization, monochloramine, point-of-use filters, and UV light. These measures have been tested over the last 30 years and are effective at controlling the growth of *Legionella*, all of them presented advantages and disadvantages that must be carefully considered [10,16].

Different studies have focused in the last years on the role of oxidizing agents, notably hydrogen peroxide (H_2_O_2_), as disinfection treatments. The use of H_2_O_2_ as a biocide is widespread, and it is increasingly used as a general surface disinfectant in the medical, food, and industrial fields, as well as for water treatment [21,22]. H_2_O_2_ is completely soluble in water and is stabilized in commercial formulation for disinfection treatment. It is compatible with different pipeline materials, and does not react with the organic constituents in the water to form dangerous residues with respect to chlorine, sodium hypochlorite (NaOCl), and monochloramine treatment. H_2_O_2_ decomposes rapidly in different environmental conditions due to microbial catalase and peroxidase, and other than abiotic action, the decomposition is promoted by heavy metal, oxidative, and reductive reactions. It shows a broad antimicrobial spectrum and has been shown to be active against bacteria, yeast, fungi, viruses, spores, proto-, and metazoans [23,24,25]. 

A disadvantage of using H_2_O_2_ is that its potency is influenced by several factors: pH, temperature, or the presence of substances that hamper its reactivity [26]. Since H_2_O_2_ is a renowned disinfectant, legislation [27] allows its use for the disinfection of water and in food; additionally, this compound is generally considered to have low eco-toxicity, as well as no odor or color [23,28].

To enhance its activity, H_2_O_2_ is sometimes used in combination with other oxidants such as ozone, Ag^+^, or UV radiation [24]. Silver, a biologically non-essential metal, has been investigated and used as a biocide for many years [29], and multiple strategies have been proposed for its use to treat drinking water [30,31,32]. Indeed, the World Health Organization (WHO) allows its use in drinking water. It is thought that concentrations up to 50 µg/L (ppb) in drinking water pose no risk to health [33].

The literature contains several accounts of the properties, germicidal effectiveness, and potential uses for stabilized H_2_O_2_ in healthcare facilities [34,35,36,37]. In 2015, Martin et al. [24] have demonstrated that Huwa-San peroxide (HSP), a new generation peroxide stabilized with ionic silver and suitable for continuous disinfection of potable water, preferentially interacts with the bacterial cell surface in a mechanism likely mediated by silver. Furthermore, treatment of hospital hot water systems with various formulations of H_2_O_2_/Ag^+^ compounds prevents contamination by *Legionella* and other microorganisms because of its bactericidal properties [38,39,40]. 

The H_2_O_2_/Ag^+^ formulation is stable at high temperatures, and its disinfection power increases significantly as water temperature increases. In a hot water system, a temperature range of 40–50 °C and a residual disinfectant concentration of 20–25 mg/L, seems to be able to induce a *Legionella* control [41,42]. Casini et al. suggested therefore, how a continuous feed rate of approximately 25 mg/L, was able to control the planktonic population, and silver can be deposited on the piping system, promoting a bacteriostatic effect [42].

Different commercial formulations based on H_2_O_2_/Ag^+^ are available to control *Legionella* contamination, but many studies lack data about the hospital settings and long-term applications.

Our study evaluated the effectiveness of a new disinfectant, Water Team Process 828 (WTP 828), based on H_2_O_2_ and Ag^+^ salts in the hot water distribution networks at Maria Cecilia Hospital (MCH), Cotignola (RA), Italy, controlling *Legionella* contamination.

The hospital is comprised of three buildings connected to each other but were built and submitted to renovation works at different times. The plumbing system comprises a single cold water supply and three different hot water return lines. These characteristics permitted us to study the activity of WTP 828 as three separate hot water networks (HWNs), modulating the dosage with respect to the level of *Legionella* contamination found. *Legionella* level in response to disinfection treatment was also studied by taking into account the following water network characteristics: building area, annual water consumption, hospital activities involving the use of water, that can influence the *Legionella* contamination and the disinfectant exposure to distal outlets [16]. The isolates were typed using an agglutination and genotyping approach to assess the distribution of strains in the buildings. The effect of WTP 828 was also tested on *Pseudomonas aeruginosa* (*P. aeruginosa*), one of the main components of biofilm [43], and HPC at 36 °C, commonly used as an indicator of water quality, and to monitor the effectiveness of disinfection treatment [33,44].

The physical and chemical parameters were also measured during implementation of WTP 828 treatment in all buildings in order to maintain the water quality characteristics [45,46] and preserve the plumping system materials. 

The purpose of the study is to perform an extended investigation on the effect of H_2_O_2_/Ag^+^ treatment in a complex hospital water network system. The goal is to control *Legionella* infections throughout a risk assessment model based on the use of a low-cost disinfectant, easy to dose, and less aggressive on the material pipelines, with quick and safe monitoring of residual concentrations at distal outlets. This model associated to ordinary and extraordinary maintenance procedures (e.g., flushing, temperature control, and cleaning activities) could be extended to other hospitals, companies, and leisure facilities, where water represents a risk for public health.

## 2. Results

### 2.1. Legionella Contamination 

The following results were obtained during the two phases of the study according to Italian Guidelines for prevention and control of legionellosis [7] that take into account the concentration of bacteria in relation to four levels of risk (<100, 101–100, 1001–10000, >10000 UFC/L) and the number of positive on the total number of samples collected. The measures to apply, in order to contain the risk, are different if the percentage of positive samples are <20% or >20%. 

*WTP1 Phase (October 2013 to March 2015)*

The data were obtained from the analysis of 53 hot water samples spread throughout the three buildings. We observed different *Legionella* contamination trends in the MCH buildings (Table 1): 16/25 positive samples (64.0%) in Building 1, 13/23 positive samples (56.5%) in Building 2, and 3/5 positive samples (60.0%) in Building 3. The WTP1 phase was also compared with *Legionella* contamination data collected during the previous disinfection treatment involving the ClO_2_ mixture. Although we observed a change in the percentage of *Legionella*-positive samples in MCH (Building 1-2-3) from 95.0% to 60.0%, no statistical differences were observed in terms of *Legionella* contamination levels following the introduction of WTP 828. The analysis of *Legionella* contamination inside each building revealed a significant change in the mean *Legionella* levels only in Building 2 (*p* = 0.045) (Table 1).

*WTP2 Phase (September 2014 to October 2015)*

In the second phase of the study, we observed a reduction in terms of the percentage of *Legionella*-positive samples in MCH with respect to the WTP1 phase (from 60% to 35.8%) (Table 1). The same trend was also observed for the *Legionella* contamination levels (*p* = 0.0001). The results inside each building show a marked reduction in the percentage of *Legionella*-contaminated sites in Building 2 (from 56.5% to 7.0%) and Building 3 (from 60.0% to 34.0%); by contrast, the percentage of positive samples was only slightly reduced in Building 1 (from 64.0% to 58.1%). 

The *Legionella* contamination levels displayed a significant difference between the WTP1 and WTP2 phases for Building 2 (*p* = 0.046) and Building 3 (*p* = 0.048) (Table 1). No statistical difference between phases was observed for Building 1, in which *Legionella* contamination levels of 1000 CFU/L were detected and, in accordance with Italian Guidelines [7], two shock treatments, increasing up to 50–60 mg/L of WTP 828, were performed (from February to March and from July to August 2015), resulting in a concentration of 25–30 mg/L at distal outlets. 

The data collected from the WTP2 phase were also compared with *Legionella* contamination data obtained during disinfection with the ClO_2_ mixture. The comparison revealed significant differences for Buildings 2 (*p* = 0.0001) and 3 (*p* = 0.045) and no significant differences for Building 1 (Table 1).

During the study, the water reserves, softener, and tap water output sites were *Legionella*-free (below the detection limit of the culture technique used, i.e., 50 CFU/L).

To study the risk of *Legionella* disease that could be derived from the approach used during WTP 828 treatment (WTP1 and WTP2 phase) with the outcomes after ClO_2_ mixture treatment, we used two measures of risk provided by epidemiological studies: OR in a retrospective approach, and RR in a prospective study. Comparison of the ClO_2_ mixture with WTP1 (OR, 0.3) indicated that WTP 828 was not particularly effective in any of the MCH buildings (*p* = 0.048); however, comparison of the ClO_2_ mixture with WTP2 (OR, 15.44) revealed a significant improvement in *Legionella* control with respect to the latter (*p* = 0.0001). A prospective study indicated that the level of contamination during the WTP1 phase was higher than during the WTP2 phase (RR, 0.36, *p* = 0.002), showing a decrease in *Legionella* risk (Table 2).

### 2.2. Legionella Typing 

The isolates from the WTP1 and WTP2 phases were serotyped and genotyped using standard techniques. The agglutination test permitted us to identify *L. pneumophila* serogroup 1 (SG1) and two *Legionella* species in 138/349 positive samples (39.0%). The SBT method assigned ST1 and ST104 to *L. pneumophila* SG1 isolates in 74/138 (53.6%) of the samples, while *mip* gene sequencing identified, *L. anisa* and *L. rubrilucens*, in 35/138 (25.3%); the remaining 29/138 (21.0%) samples contained a mixture of the previously described strains.

The results revealed that each HWN building was colonized by a different mixture of *Legionella* spp. Accordingly, Building 1 isolates were the most diverse with *L. pneumophila* SG1 (ST1 and ST104) and *L. species* (*L. anisa* and *L. rubrilucens*). All Building 2 isolates belonged to *L. pneumophila* SG1 (ST1 and ST104), and Building 3 samples demonstrated the presence of *L. pneumophila* SG1 (ST1), with some samples containing a single *L. species* strain (*L. anisa* or *L. rubrilucens*). 

The serotyping and genotyping data about the bacterial concentration ranges (log_10_ CFU/L) are presented in Table 3.

During the study period, no significant association was found between *Legionella* colonization in the buildings and specific serogroups or strains. However, in Building 1, as after the two shocks treatment (February–March 2015 and July–August 2015), we observed a decrease in *L. pneumophila* SG1 levels, and detection of the other species, mainly *L. anisa* and *L. rubrilucens*. More experiments are still in progress.

### 2.3. Pseudomonas aeruginosa and HPC Typing

During the previous treatment by ClO_2_ mixture, the risk assessment plan for *Legionella* surveillance was performed without control of HPC at 36 °C and *P. aeruginosa*. After the introduction of WTP 828 treatment, during the study period (WTP1 and WTP2 phase), 349 hot water and 65 cold-water samples distributed among distal outlets, water reserves, softener, and tap water outputs of MCH were also analyzed for the presence of *P. aeruginosa* and HPC at 36 °C. 

*P. aeruginosa* was not detected (as prescribed in D. Lgs 31/2001) [46] in either cold or hot water samples. 

The HPC at 36 °C results for each building expressed as the mean concentration ± SD (log_10_ CFU/mL) were as follows: 0.82 ± 0.25 for Building 1 (0.48–1.20 log_10_ CFU/mL), 0.77 ± 0.65 for Building 2 (0.30–0.90 log_10_ CFU/mL), and 0.94 ± 0.35 (0.48–1.11 log_10_ CFU/mL) for Building 3. 

At all sites, the contamination range was lower than the D. Lgs 31/2001 [46] limit of 20 CFU/mL (1.3 log_10_ CFU/mL).

### 2.4. Physical and Chemical Parameters of Water

The physical and chemical parameters linked to the quality of water after disinfection with WTP 828 were measured only during the WTP2 phase when relevant changes were made to the risk assessment plan. A total of 296 hot water and 65 cold water samples were analyzed. Physical and chemical data related to previous disinfection treatments and to the WTP1 phase are not reported because the *Legionella* surveillance during these phases took into account only bacteriological parameters. 

The hardness, turbidity, and conductivity of the water (all of which are associated with the release of iron and total phosphorus) in the cold and hot water systems were not affected by WTP 828 treatment; these data are in agreement with recommendations established by Italian legislation [46]. In particular, the mean concentration of Ag^+^ remained lower than the detection limit (3 µg/L) and in line with WHO Guidelines for drinking water [33]. These results are shown in Table 4.

### 2.5. LD Surveillance 

During the study (WTP1 and WTP2 phases), 32 patients underwent urine antigen testing and other diagnostic tests because of suspected pulmonary signs of pneumonia. The negative results obtained confirmed the absence of cases of nosocomial legionellosis.

## 3. Discussion

In this study, the effectiveness of WTP 828 was evaluated in a MCH water system because of its unique layout (i.e., constructed as three separate buildings). The water distribution system is characterized by a single tap water output, and each building is equipped with its own hot water return line and water disinfection treatment system. Before the introduction of WTP 828, MCH implemented a disinfection approach of continuous treatment with a ClO_2_ mixture (dosage of 0.5 mg/L). This type of treatment led to corrosion of some parts of the plant and a visible decrement of the efficiency of *Legionella* colonization containment, as demonstrated by the high number of *Legionella*-positive samples in the three buildings (114/120, i.e., 95.0%) and the presence of *P. aeruginosa* in some water outlets, found during not routinely control (data not shown). In October 2013, the MCH Health Director decided to introduce WTP 828 into Building 2, as well as at available sampling points in Buildings 1 and 3. 

The results obtained were discussed, for each MCH building, in relation to the period of WTP 828 introduction, as follows:

- Building 1: WTP1 from October 2013 to December 2014 and WTP2 from January 2015 to October 2015;
- Building 2: WTP1 from October 2013 to August 2014 and WTP2 from September 2014 to October 2015. This building was not subjected to any changes in disinfectant concentration or renovation works;
- Building 3: WTP1 October 2013 to March 2015 and WTP2 from April 2015 to October 2015.

Introduction of WTP 828 during the WTP1 phase (led to an overall reduction of the percentage of *Legionella*-positive samples (from 95% to 60.0%) when compared with the ClO_2_ mixture. Preliminary results showed that WTP 828 treatment led to a marked reduction of contamination in Building 2 (*p* = 0.046); these results can be explained by the observation that Building 2 was the first building to undergo WTP 828 treatment. Additionally, this building has never been refurbished or otherwise altered since it was built. By contrast, Buildings 1 and 3 underwent an upgrade of the water distribution system and the construction of new accommodation sites, thus accounting for the small number of samples collected due to the absence of outlets.

These results are in line with Italian Guidelines and other authors [7,10], regarding the needs to have a broad knowledge of the buildings characteristics, the water distribution system, the pipelines material, and the disinfectant interaction with them, before to choose the disinfection method to use. As described by other authors, many actions undertaken during renovation works can induce a mobilization of biofilm and alter the flushing of disinfectant at distal outlets due to the lower water consumption and the closing of some outlets. The particulate and the increase of water turbidity, produced by structural works, can induce a decomposition of oxidants such as H_2_O_2_ [10,47,48]. Moreover, distal outlets in some parts of the hospital are seldom used: In particular areas of the hospital (surgeries room, intensive care units, etc.) the sterile water is preferred to the tap water, therefore the consumption of tap water is lower too. 

The conclusion of accommodation works and the completion of the final structures within Buildings 1 and 3 allowed us to implement the risk assessment plan for *Legionella* control and to increase the number of sampling sites and the frequency of sampling, according to several studies indicating that routine cultures of the hospital water supply for *Legionella* may provide an important strategy for the prevention of legionellosis outbreaks [49].

To assess the effectiveness of WTP 828, we compared data obtained during the WTP1 phase with those obtained during the WTP2 phase. We observed a reduction both in the percentage of positive samples and the mean *Legionella* levels in all buildings during WTP2. In detail, a significant reduction in the amount of *Legionella* contamination was observed in Buildings 2 and 3 (*p* = 0.001 and 0.037, respectively). *Legionella* control was then maintained for the entire duration of the study. 

The observed differences in *Legionella* colonization between the buildings can be ascribed to the different uses and water consumption in these buildings. Risk factors that should not be overlooked are, in fact, the scale of the extension, connection of existing pipes within the newly constructed branched networks, presence of dead branches, pipe characteristics (e.g., materials, age), treatment of the water system (e.g., water softening and disinfection), intended utility, and maintenance procedures [20]. In light of these considerations, we also investigated our results in relation to data concerning annual water consumption in each building size, number of water outlets, pipe materials, and the timing of renovation works. 

Building 1 has six levels and covers an area of 18,539.93 m^2^. It mainly comprises offices, surgery rooms, operating rooms, and diagnostic rooms, some of which only require the use of sterile water; therefore, overall water consumption is limited. In this building, the third floor hosts a technical room for air treatment without water outlets; therefore, some closed pipes are present. The water consumption (1913 m^3^/year) indicated a much lower use than in Building 2 (3017 m^3^/year), suggesting lower water flushing from the outlets. It is evident that low use and stagnation of water may affect the activity and delivery of disinfectant, reducing its effect on the microorganisms [50,51]. The renovation works were completed in 2015. The pipelines that made up the water network comprised mainly multilayer PVC, which increases biofilm formation [52,53,54,55,56,57]. Our data revealed that, despite a reduction in the percentage of *Legionella*-positive sites and mean *Legionella* levels, WTP 828 was not completely effective in this building, demonstrating continuous fluctuations in the amount of *Legionella* spp. colonization. Corrective measures have since been implemented; these include two chemical shock treatments as above described and the implementation of maintenance hospital procedures such as increasing the flushing time once a week and during weekends, anti-scale procedures at each distal outlet (every fifteen days), and strictly cold and hot water temperature control weekly [12]. The long-term effects of our interventions resulted in the maintenance of *Legionella* contamination levels below the range of alert prescribed by the Italian Guidelines (101–1000 CFU/L); this will limit the risk of exposure and preserve the health of patients and workers.

In Building 2, the presence of multiple outlets (336) and some facilities with high water consumption (e.g., cafes, restaurants, and markets) suggested that water flushing facilitated the circulation of the disinfectant in the plumbing system, reducing the number of bacterium-positive samples and the *Legionella* concentration, in accordance with a study by Douterelo et al. [50]. The water distribution system consists of mainly galvanized iron, which, as suggested in the literature, on the contrary to plastic material, such as polyethylene (PE) and polyvinylchloride (PVC) [54,57], together with prolonged use of the WTP 828 disinfectant, may help to inhibit *Legionella* colonization and enable the maintenance of this inhibition over long periods.

Building 3 is the smallest structure of MCH, covering an area of 1271.06 m^2^. The total annual water consumption in this building is 589 m^3^ per 129 outlets. The services (two food preparation areas) and in-patient rooms allow the daily circulation of disinfectant within the plumbing system, thereby contributing to the effectiveness of WTP 828 in controlling *Legionella* contamination levels in this building.

The impact of the disinfectant used (WTP 828 or ClO_2_ mixture) and the type of approach applied in MCH to reduce the risk of acquiring *Legionella* disease was therefore studied by calculating OR and RR epidemiological measures. Significant results were obtained comparing WTP 828 versus ClO_2_ by calculating the OR measure, which showed that the introduction of WTP 828 after replacement of the ClO_2_ mixture was a good strategy to decrease risk in MCH, increasing control of the *Legionella* contamination level. By comparing the two different phases of our study, WTP1 versus WTP2, in a prospective approach using the RR measure, showed that the approach implemented during the WTP2 phase characterized by a new monitoring plan, the increase in the number of samples and adoption of a new protocol of flushing, cleaning and disinfectant monitoring, can help to decrease the risk of acquiring the disease.

The risk of Legionellosis is linked to different factors such as personal characteristics and immunodeficiency status, but such personal risk factors can also enhance the risk of acquiring the disease when environmental control is not correctly performed or is underestimated [58,59].

The serotyping and genotyping data revealed different colonization patterns in MCH buildings, but we did not find a significant association between the presence of some *Legionella* strains in MCH buildings.

Different authors have suggested that changes in the disinfection treatment regime (e.g., the type of disinfectant) or the dose (e.g., shock treatment) might influence the type of *Legionella* strains that become prevalent [42,47,60,61,62]. In agreement with these observations, the increased WTP 828 dosage used during the shock treatment performed two times in Building 1 resulted in a reduction in *L. pneumophila* SG1 and increases in *L. species: L. anisa* and *L. rubrilucens* (data not shown). 

The absence of *P. aeruginosa* from water samples during the study period seems to indicate a good effect of WTP 828 on the containment of these bacteria with respect to previous evidence provided by the MCH Health Director. During the previous treatments with ClO_2_, the release of pipelines materials, the presence of accommodation works, and a lack of cleaning procedures favored the growth of *P. aeruginosa* (data not shown). The introduction of routine cultures of this bacterium in hot and cold-water samples suggested in the MCH study, also helped to control the efficiency of the cleaning procedures, other than evaluate the biofilm presence, where *Legionella* and other microorganisms become more resistant to antibiotics and disinfectants [48].

In detail, the protocol undertaken by the cleaning staff every fifteen days, i.e., cleaning and flushing procedures (e.g., disinfecting the taps and showers and flushing cold and hot water outlets) played an important role in preventing biofilm formation [63,64], which can support *Legionella* growth. Semiannual meetings with the stakeholders and hospital staff to inform them of the bacterial infection risk and the procedures undertaken to reduce such risks were also useful.

HPC is an indirect indicator of water quality and is often used to assess the efficacy of water treatment and to measure the amount of heterotrophic bacteria colonization in distribution systems. Despite some studies showing the absence of a correlation between levels of HPC bacteria and human infection, suggesting that HPC levels are not highly predictive of *Legionella* colonization, the control of this parameter could help to understand whether the water system contains potentially infectious organisms [33,44]. Our results indicated that WTP 828 performed well with respect to HPC containment during the entire study period, maintaining levels below directive limits [46].

A weakness of this study is that we were unable to demonstrate that WTP 828 treatment did not affect the physical and chemical parameters of the water in all study periods. Data regarding previous disinfection treatment with the ClO_2_ mixture, along with data from the WTP1 phase, were missing; therefore, we could not compare changes in water quality that occurred during all study periods, underscoring the important role played by environmental monitoring of physical and chemical parameters when demonstrating the efficacy of a disinfectant. By contrast, the control of these parameters during the WTP2 phase allowed us to monitor the effect of the disinfectant on water pipes, take measures to prevent damage to the water network, and maintain the quality of “drinking water” to prevent risks to human health. 

According to Borella et al. [10], the choice of WTP 828 has been carried out also on a careful evaluation of a cost-effective analysis, considering the system of disinfectant production (pump or others), the maintenance costs (disinfectant provision, service, etc.) and the potential dangerous effect on water pipelines, other than the possibility to safety measure disinfectant residues to outlets by colorimetric strips by staff. 

## 4. Materials and Methods 

### 4.1. MCH Structure and Water Outlet Characteristics

This study was conducted at Maria Cecilia Hospital (MCH), an Italian hospital founded in 1973 located in Cotignola (RA, Emilia Romagna). 

The structure of the hospital is complex as it comprises three separate buildings (Buildings 1–3) built during different years and covering a total area of 27,989.64 m^2^ (Supplementary Figure S1). Buildings 1 and 3 were constructed in 2001 and subjected to renovation or enlargement works until 2015. Building 2 is the main MCH building and did not undergo any changes in its structure during the whole study period.

These characteristics permit the study of WTP 828 activity as three separate HWNs and allow modulation of the dose with respect to the level of *Legionella* contamination, water demand, intended use, and renovation works (Supplementary Table S1).

At the end of the renovation works, the final structure of MCH had 122 in-patient rooms, each with one or two beds and an en-suite bathroom. There were 212 beds in total, mainly located in Building 2, and 769 water outlets (e.g., taps and showers) located in in-patient rooms, communal areas, diagnostic and operating rooms, offices and services, as follows:

- Building 1 covers an area of 18,539.93 m^2^ and has six floors with communal areas for the guests (e.g., bar, restrooms), operating rooms, outpatient services (diagnostic and consulting rooms), intensive care units, and 27 in-patient rooms located on the second floor. In this building, 21 sampling points and one hot water return line point were identified. Two of the 21 sampling points in in-patient rooms were monitored monthly, on a rotational basis by room number (Supplementary Table S1).
- Building 2 covers an area of 8178.68 m^2^, with six floors with 70 in-patient rooms distributed on floors one to four. Twenty-two sampling points were monitored (21 plus one hot water return line point) in this building, ten of which were in in-patient rooms, which were monitored monthly and rotated by room number (Supplementary Table S1).
- Building 3 covers an area of 1271.06 m^2^ and was recently expanded, with a complete renovation in February 2015. The building has six floors, with 25 in-patient rooms located on the third and fourth floors. Due to their size and comfort, these rooms are designated as “suites” and are reserved for long-term guests. There were 13 sampling points (and one hot water return line point) in this building, six of which were in in-patient rooms, which were monitored monthly and rotated by room number (Supplementary Table S1).

### 4.2. Hospital Water Network (HWN)

The hospital plumbing system is very complex, partially antiquated, and (depending on age built) predominantly made up of galvanized iron and polyvinylchloride (PVC) multi-layers. The HWN was coated with an anti-scale treatment to create a protective film on the galvanized iron and PVC surface, as suggested by WHO guidelines in 2011 [32]. It consists of a product based on natural mineral salts such as orthophosphates, polyphosphates, and alkaline silicates dosed at 0.1 mg/L. The MCH structural characteristics, material pipelines, and water consumption for each building were kindly provided by Health Direction and described in Supplementary Table S1. 

**Figure 1:**
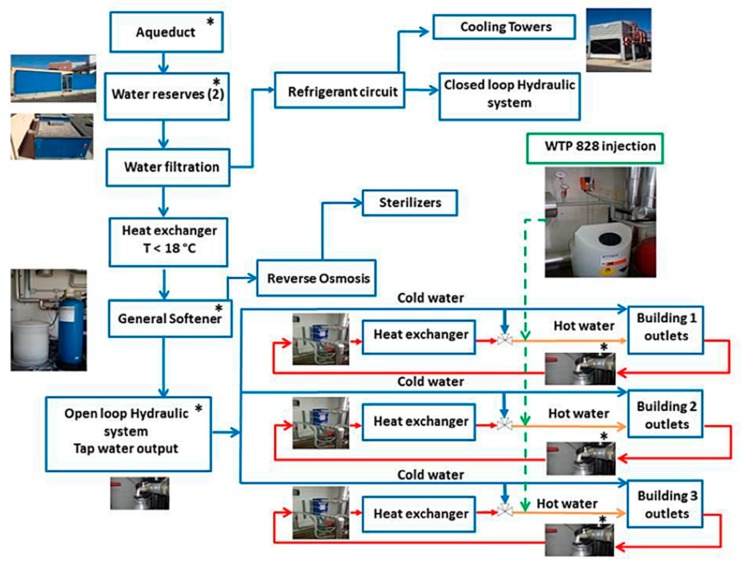
The scheme of the MCH water network with main sampling points in technical rooms (*).

All buildings are supplied with the same municipal water aqueduct, which brings water from the Ridracoli dam located 53 km from Cotignola. The water is first collected in two 30 m^3^ water reservoirs outside the buildings. After filtration through a 150 µm pore size filter, water is fed into two pipelines: one to the cooling towers and refrigerant circuit (a closed loop hydraulic system) and the other to the water treatment station (an open loop hydraulic system). A plan of the water distribution network is shown in Figure 1.

A heat exchanger maintains the temperature of the cold water in the treatment station at <18 °C; the hardness of cold water is treated with a general softener to reduce its value between 12–15 °f (water moderately hard), which is in line with Italian and European Council directives [45,46]. Some of this water supplies the sterilizers after reverse osmosis treatment, and another portion is used as cold water by the hospital. The cold water is distributed to the substations within each building through a single tap water output. Three different heat exchangers (one at each substation) produce hot water. The cold and hot water circuits are independent of one another, and each building has its own hot water return line.

### 4.3. WTP 828

Water Team Process 828 (WTP 828) developed by an Italian Company involved in disinfectant production (Water Team S.r.l., Forlì (FC), Italy) is a multi-component oxidizing biocide formulated using a stabilized combination of H_2_O_2_ (34%, wt/wt) and Ag^+^ salts (0.003%, wt/wt) in demineralized water, resulting in a highly effective disinfection solution. The formulation is covered by Italian regulation on intellectual property rights and actually is under investigation to acquire a patent. It is licensed by European and Italian legislation [27,65] for its application in drinking water. The synergistic action of H_2_O_2_ and Ag^+^ salts renders the biocide more powerful than H_2_O_2_ alone [66,67]. Ag^+^ was used to increase the activity of the peroxide, and Ag^+^ forms an insoluble salt at distal points and is able to attach to pipes and exert bacteriostatic effects on biofilms [41,68]. 

The WTP 828 is injected into mixed water (hot/cold) after hot water output downstream from the heat exchangers and dosed proportionally to the volume of water supply.

WTP 828 was introduced into MCH for the first time in October 2013 after replacement of a previous disinfection system based on a continuous treatment performed with chlorine dioxide (ClO_2_ mixture) at a dosage of 0.5 mg/L. This treatment, which was used from September 2009 to September 2013, had compromised the water pipelines and corroded some parts of the plant, thereby reducing efficacy with respect to *Legionella* colonization and supporting the presence of *P. aeruginosa* in some water outlets.

The WTP 828 concentrations during the study were modulated according to the microbiological results for each building. In particular, the initial dose of 30 mg/L resulting in a final concentration of 5–10 mg/L at distal outlets remained the same in Buildings 2 and 3 throughout the whole study period. By contrast, two shock treatments were required in Building 1 (from February to March and July to August 2015); at these times, the injected dose of WTP 828 increased up to 50–60 mg/L, which resulted in 25–30 mg/L of H_2_O_2_ at the distal outlets.

### 4.4. Study Design

This study was conducted in two experimental phases designated WTP1 and WTP2 in relation to the timing of the introduction of WTP 828, the renovation or enlargement works conducted in the buildings and the acquisition of a new risk assessment plan. The data collected during the WTP1 and WTP2 phases were then compared to evaluate differences in the efficacy of the WTP 828 treatment in the HWNs of the three buildings.

These data were then compared with the data obtained during disinfection with the ClO_2_ mixture (i.e., ClO_2_ mixture versus WTP1 phase and ClO_2_ mixture versus WTP2 phase) to assess the effects of WTP 828 on *Legionella* contamination.

The details of the study period for each building are described below:

- Building 1: WTP1 from October 2013 to December 2014 and WTP2 from January 2015 to October 2015;
- Building 2: WTP1 from October 2013 to August 2014 and WTP2 from September 2014 to October 2015. This building was not subjected to any changes in disinfectant concentration or renovation works;
- Building 3: WTP1 October 2013 to March 2015 and WTP2 from April 2015 to October 2015.

During the WTP1 phase, disinfection with WTP 828 started in Building 2 in October 2013 and in some locations within Buildings 1 and 3, which were under construction or undergoing expansion in this period.

Sampling of hot water systems was performed according to the risk assessment plan, which was approved by the MCH Health Director and the Local Authority. There were 29 sampling points spread throughout the three buildings among consulting and diagnostic rooms, wards, common areas, and in-patient rooms, which were monitored every four months on a rotational basis. During this phase, a total of 53 samples were subjected to microbiological analysis for detection of *Legionella, P. aeruginosa* and heterotrophic plate count (HPC) bacteria at 36 °C, and data were collected.

During the WTP2 phase, renovation works of Buildings 1 and 3 were completed (January 2015 and April 2015, respectively). WTP 828 treatment was extended to all parts of these buildings and, based on preliminary results regarding WTP 828 efficacy (WTP1 phase), a new risk assessment and monitoring plan were adopted.

In accordance with Italian Guidelines [7], sampling points were chosen at the following three locations: in the vicinity of, mid-way to, and away from the technical room. The location of the sampling points took into account the size of the building, the number of in-patient rooms, the health services provided, the risk of patient, and worker exposure to bacteria and epidemiological data. 

Every month, samples were collected from the technical room: one from the aqueduct, two from the cold water reserves, one downstream of the general softener treatment, one from a tap water output, and three from the hot water return lines (1a for Building 1, 1b for Building 2, and 1c for Building 3), and from another 55 sampling points in offices, consulting and diagnostic rooms, wards, common areas, and in-patient rooms (63 points in total). Despite the large number of in-patient rooms, the alternating sampling method enabled sampling of almost all in-patient rooms in the three buildings.

The increased time of monitoring (from once every four months to monthly), extension of the disinfection treatment, and development of a final MCH structure permitted the study of the modulation of microbiological and physical-chemical parameters in a total of 296 hot water and 65 cold water samples.

### 4.5. Sample Collection and Microbiological Analysis

Hot water and cold water (2 L) were collected in post-flushing modality (running water for 1 min) in sterile polytetrafluoroethylene (PTFE) bottles containing a sodium thiosulfate solution (10%, v/v). Microbiological analyses were performed in accordance with ISO11731:2017 [69] to detect and enumerate *Legionella*. During *Legionella* surveillance, according to Italian Guidelines [7], the level of risk took into account the concentration of bacteria and percentage of positive samples. 

Samples were concentrated using 0.22 µm polycarbonate pre-sterilized filter membranes (Sartorius Stedim Biotech, Göttingen, Germany). 

The concentrated samples (filtered, F) were then heated (for 30 min at 50 °C) to inhibit interfering microbiota (heated, H). Then, 0.1 mL of the untreated sample (UN) and 0.1 mL of each F and H sample were spread in duplicate onto GVPC agar plates (*Legionella* GVPC selective medium, Thermo Fisher Scientific, Oxoid Ltd., Basingstoke, UK), and incubated at 35.5 °C in a humid (2.5% CO_2_) environment. 

The plates were examined after four, eight, and 14 days, and colonies with a typical *Legionella* morphology (presumptive) were enumerated and confirmed by sub-culture on BCYE agar with and without cysteine. The isolates that grew on BCYE but failed to grow on the cysteine-free medium were verified serologically by an agglutination test (*Legionella* latex test kit; Thermo Fisher Scientific, Oxoid Ltd.). The data are expressed as the mean concentration ± standard deviation (SD) of the log_10_ colony forming units (CFU) per liter of water (log_10_ CFU/L) including all samples analyzed (positive + negative). The detection limit of the culture technique was 50 CFU/L. The samples with a value of <50 CFU/L were considered negative according to ISO 11731:2017 [69].

Other microorganisms can affect the growth of cultivable *Legionella*, and the samples were simultaneously analyzed for the presence of *P. aeruginosa*, a known competitor of *Legionella* that inhibits its growth on medium [70]. The analyses were performed according to UNI EN ISO 16266:2006 [71] using a selective Pseudomonas agar (Biolife, Milan, Italy). The detection limit of the culture technique was 1 CFU/100 mL.

The heterotrophic plate count (HPC) at 36 °C was used as an indicator of the actual level of bacterial contamination at the sampling points. The HPC is a useful indicator of increased microbial growth, increased biofilm activity, extended retention times, water stagnation, or breakdown of the integrity of the system [33,72]. The analyses were performed using a standard plate method based on tryptic glucose yeast agar (Biolife) in accordance with UNI EN ISO 6222:2001 [73]. The data are expressed as the mean concentration ± SD of the log_10_ CFU per milliliter of water (log_10_ CFU/mL) including all samples (positive + negative). 

The detection limit of the culture technique was 1 CFU/mL.

### 4.6. Legionella Typing

Colonies identified by the agglutination test as belonging to the genus *Legionella* were subsequently analyzed by DNA sequencing. In particular, all strains identified as *L. pneumophila* were analyzed by sequence-based typing (SBT) to determine the sequence type (ST); strains identified as *Legionella* species were analyzed by *mip* sequencing. Genomic DNA was extracted from cultures using the InstaGene Purification Matrix (Bio-Rad, Hercules, CA, USA). SBT was performed according to an ELDSNet protocol (http://bioinforatics.phe.org.uk/legionella/legionella_sbt/php/sbt_homepage.php). The protocol was based on the sequencing of seven genes (flaA, pilE, asd, mip, mompS, proA, and neuA) and on the assignment of a ST allelic profile by the ELDSNet database (http://www.hpabioinformatics.org.uk/cgibin/legionella/sbt/seq_assemble_legionella1.cgi). 

The strains that were serotyped by agglutination as *L. species* were then genotyped by *mip* gene amplification via the polymerase chain reaction (PCR) using degenerate primers, as described in 1998 by Ratcliff et al. [74] and modified by M13 tailing to avoid noise in the DNA sequence [75]. Gene amplification was carried out in a 50 µL reaction volume containing DreamTaq Green PCR Master Mix 2x (Thermo Fisher Scientific, OxoidLtd., Basingstoke, UK) and 40 pmol of each primer; 100 ng of DNA extracted from the presumptive colonies of *Legionella* was added as template. The same amounts of DNA from *Legionella pneumophila* (*L. pneumophila*) type strain EUL00137 provided by the European Working Group for *Legionella* Infections [76] and fetal bovine serum were used as positive and negative controls, respectively. 

Following purification, DNA was sequenced using BigDye Chemistry and analyzed on an ABI PRISM 3100 Genetic Analyzer (Applied Biosystems, Foster City, CA, USA). Specifically, *mip* amplicons (661–715 bp) were sequenced using M13 forward and reverse primers (M13 FW, 5′-TGTAAAACGACGGCCAGT-3′; M13 RW, 3′-CAGGAAACAGCTATGACC-5′) to obtain complete coverage of the sequenced region of interest. Raw sequencing data were assembled using CLC Main Workbench 7.6.4 software (https://www.qiagenbioinformatics.com/). The sequences were compared with sequences deposited in the *Legionella mip* gene sequence database using a similarity analysis tool (http://bioinformatics.phe.org.uk/cgi-bin/legionella/mip/mip_id.cgi). The identification at the species level was conducted based on 98% similarity to a sequence in the database [77].

### 4.7. Physical and Chemical Parameters of Water

The physical and chemical parameters of water were analyzed only during the WTP2 phase, before this phase the hospital did not have any data on water quality as prescribed by WHO [49]. 

Cold water samples (1 L) were collected from each of the following locations: the aqueduct, water reserves, softener, and tap water output. Hot water samples (1 L) were collected from each of the three hot water return lines and distal outlets. The pH, hardness (°f), conductivity (µS/cm), turbidity (nephelometric turbidity units), total iron content (mg/L), total phosphorus content (mg/L of P_2_O_5_), and Ag^+^ content (µg/L) were monitored monthly during the session sampling.

The analysis of total iron and phosphorus content (orthophosphate, condensed phosphate, and organic phosphate) allowed us to monitor the maintenance of anti-scale and corrosion treatment.

Temperature (°C) and residual WTP 828 levels [the peroxide component (mg/L)] were measured and recorded at distal outlets weekly in each building. WTP 828 (peroxide component) was measured using an MQuant™ Peroxide Test (Merck KGaA, Darmstadt, Germany) according to the manufacturer’s instructions. 

Other parameters were measured using different techniques according to standardized APAT CNR IRSA methods [78].

In our study the disinfection treatment was performed by a disinfectant based on H_2_O_2_/Ag^+^, therefore the dosage of DBPs release in water is not necessary. The chemical water compounds measured are listed in Table 4. The results are expressed as the mean value ± SD.

### 4.8. Data Analyses

Bacteriological data were converted into log10 x values to normalize the distribution for the correlation analysis. The normality of continuous variables was assessed using the Shapiro-Wilk test, and data are presented as the mean ± SD. Continuous variables were evaluated using one-way ANOVA and a post-hoc test (Bonferroni), and categorical variables were compared using the χ2 and Mann Whitney test. One-way ANOVA and the post-hoc test (Bonferroni) were conducted to assess differences between disinfectant treatments and between buildings.

To test the changes in *Legionella* risk between treatments, we used odds ratios (ORs) in a retrospective analysis and relative risk (RR) in a prospective analysis. In detail, OR was calculated for WTP1 versus the ClO_2_ mixture and WTP2 versus the ClO_2_ mixture, and RR was calculated for the prospective treatments (WTP2 versus WTP1). Statistical analyses were performed using STATA version 10.0 (Stata Corp., College Station, TX). A *p*-value < 0.05 was accepted as significant.

### 4.9. Hospital LD Surveillance

MCH performed active legionellosis surveillance beginning in 2013. Data were collected throughout the entire study period (WTP1 and WTP2 phases). The symptoms of legionellosis are consistent with an acute infection of the lower airways, with clinical and/or radiological signs of focal pneumonia. A preliminary diagnosis was routinely confirmed by a urine antigen test (*Legionella* Urine Antigen EIA, Biotest, Milan, Italy) and a serological immunofluorescence test (*L. pneumophila* IFA, Meridian Diagnostic Europe, London, UK).

## 5. Conclusions

It is often difficult to guarantee the absence of *Legionella* from water distribution systems, even if a disinfection system is in place. Our data revealed that differences in three buildings belonging to the same structure were linked to building size, water consumption rates, the number of outlets, and their intended use. WTP 828 performed well in terms of reducing *Legionella* contamination, but only a change in the study approach (adequate risk assessment plan, increase in monitoring samples sites, and alteration of the WTP 828 dosage in relation to the *Legionella* levels) facilitated the discovery of differences in *Legionella* colonization and an understanding of disinfectant activity dynamics.

Further investigations are needed to elucidate how the dose of disinfectant affects the presence of specific strains in each building and to generate a risk map highlighting the phylogenetic correlations between strains. The assessment of changes in colonization dynamics will be useful for controlling the concentration and type of disinfectant that can be used in a water system (i.e., shock or continuous treatment, bacterial resistance development) in relation to accommodation works and technical operations in the water network that could support *Legionella* proliferation. 

The low cost of WTP 828, the dosage by a pump, the easy maintenance procedures and simple and safe check of disinfectant residue at distal outlets, suggest that the approach used in this study could be a valid alternative to traditional disinfection methods.

## Figures and Tables

**Table 1:** *Legionella* concentration in three buildings of Maria Cecilia Hospital (MCH) for each study phase.

	Treatment/Study Phase	Number of Samples	Number of *Legionella* Positive Samples (%)	Mean *Legionella* Levels (log_10_ CFU/L) ± SD	Comparison between Phases	*p* Value
**MCH (Buildings 1–3)**	ClO_2_ mixture	120	114 (95.0)	2.54 ± 0.74	WTP1 *vs.* ClO_2_	1
	WTP1	53	32 (60.0)	2.43 ± 0.95	WTP1 *vs.* WTP2	0.0001 *
	WTP2	296	106 (35.8)	1.67 ± 0.66	WTP2 *vs.* ClO_2_	0.0001 *
**Building 1**	ClO_2_ mixture	47	46 (98.0)	2.47 ± 0.67	WTP1 *vs.* ClO_2_	0.623
	WTP1	25	16 (64.0)	2.80 ± 0.87	WTP1 *vs.* WTP2	0.060
	WTP2	141	82 (58.1)	2.21 ± 0.55	WTP2 *vs.* ClO_2_	0.835
**Building 2**	ClO_2_ mixture	58	53 (91.3)	2.39 ± 0.63	WTP1 *vs.* ClO_2_	0.045 *
	WTP1	23	13 (56.5)	1.81 ± 0.82	WTP1 *vs.* WTP2	0.046 *
	WTP2	108	8 (7.0)	1.26 ± 0.40	WTP2 *vs.* ClO_2_	0.0001 *
**Building 3**	ClO_2_ mixture	15	15 (100.0)	3.12 ± 1.04	WTP1 *vs.* ClO_2_	1
	WTP1	5	3 (60.0)	2.97 ± 0.71	WTP1 *vs.* WTP2	0.048 *
	WTP2	47	16 (34.0)	1.47 ± 0.60	WTP2 *vs.* ClO_2_	0.01 *

* Values are statistically significant at *p* < 0.05.

**Table 2:** Odds ratio and relative risk during the study phases.

**Study phases**	**Odds Ratio (OR)**	**95% Confidence Intervals**	***p* value**
ClO_2_ mixture versus WTP1	0.30	0.09–1.02	0.048 *
ClO_2_ mixture versus WTP2	15.44	5.14–46.33	0.0001 *
**Study phases**	**Relative Risk (RR)**	**95% Confidence Intervals**	***p* value**
WTP1 versus WTP2	0.36	0.18–0.71	0.002 *

* Values are statistically significant at *p* < 0.05.

**Table 3:** Serotyping and genotyping of *Legionella* isolates in MCH buildings (WTP1 and WTP2 phases).

Building	Positive Samples	Serotyping	Genotyping	*Legionella* Isolates WTP1 Versus WTP2	Isolates/Positive Samples		Range of *Legionella* Concentration log_10_ CFU/L (Min–Max) *
Building 1	98	*L. pneumophila* SG1	ST1 and ST104	WTP1	36/98		<1.70–5.80
		*L. species*	*L. rubrilucens*	WTP2	11/98		<1.70–4.60
		*L. species*	*L. anisa*	WTP2	20/98		<1.70–3.77
		*L. species*	*L. rubrilucens + L. anisa*	WTP2	2/98		2.83–3
		*L. pneumophila* SG1 + *L. species*	ST1 and ST104 + *L. rubrilucens*	WTP2	29/98	9/29	2–5.69
			ST1 and ST104 + *L. anisa*	WTP2		20/29	<1.70–3.53
Building 2	21	*L. pneumophila* SG1	ST1 and ST104	WTP1 and WTP2	21/21		<1.70–4.50
Building 3	19	*L. pneumophila* SG1	ST1	WTP1	17/19		<1.70–4.18
		*L. species*	*L. rubrilucens*	WTP2	2/19	1/19	2.10
			*L. anisa*	WTP2		1/19	1.70

* (<1.70 Log UFC/L, detection limit).

**Table 4:** WTP2 phase: Physical and chemical parameters of water in MCH aqueduct and distal outlets.

Parameters	Standardized Methods	Principle of the Method	U.M.	Sampling Points (Mean Value ± SD)						
				Aqueduct (n = 13)	Building 1		Building 2		Building 3	
					Hot Water Return Line (1a) * (n = 13)	Distal Outlets (n = 128)	Hot Water Return Line (1b) * (n = 13)	Distal Outlets (n = 95)	Hot Water Return Line (1c) * (n = 3)	Distal Outlets (n = 34)
**pH**	APAT IRSA CNR 2060 Man 29 2003	Potentiometric method		7.82 ± 0.23	7.88 ± 0.24	7.95 ± 0.07	7.87 ± 0.27	7.92 ± 0.04	7.95 ± 0.19	7.94 ± 0.09
**Hardness**	APAT IRSA CNR 2040 Man 29 2003	Complex metric titration	° f	12.10 ± 4.28	12.13 ± 3.14	11.07 ± 3.46	12.63 ± 3.50	9.75 ± 4.44	12.15 ± 2.44	11.92 ± 3.05
**Conductivity**	APAT IRSA CNR 2030 Man 29 2003	Conductimetric method	µS/cm	407.71 ± 35.20	416.53 ± 41.80	374.91 ± 39.97	420.00 ± 40.70	447 ± 13.78	423.44 ± 29.75	424.80 ± 32.09
**Turbidity**	APAT IRSA CNR 2110 Man 29 2003	Spectrophotometric method	NTU	0.40 ± 0.09	0.39 ± 0.12	0.63 ± 0.22	0.52 ± 0.25	1.69 ± 1.41	0.88 ± 0.97	0.94 ± 0.63
**Total iron**	APAT IRSA CNR 3160A Man 29 2003	Flame atomic absorption spectroscopy (FAAS)	mg/L	<0.04	0.04	0.05 ± 0.03	<0.04	<0.03	0.03	0.09 ± 0.10
**Total phosphorus**	APAT IRSA CNR 4060 Man 29 2003	Spectrophotometric method	mg/L P_2_O_5_	<0.2	3.19 ± 1.31	3.05 ± 0.49	3.40 ± 1.40	3.52 ± 0.76	1.68 ± 0.85	2.05 ± 0.59
**Silver**	EPA Method 272.2	Electrothermal atomization atomic absorption spectrometry (ETA-AAS)	µg/L	<3	<3	<3	<3	<3	<3	<3
**Temperature**	EPA Method 170.1	Thermistor probe	°C	15.20 ± 3.40	49.03 ± 2.42	49.75 ± 2.30	50.20 ± 0.61	50.38 ± 1.80	53.87 ± 4.13	51.07 ± 2.25
**Peroxide**	Peroxide Test MQuant ™		mg/L	not detected	7.42 ± 2.71	15.0 ± 7.07	8.46 ± 3.15	14.72 ± 4.75	5.83 ± 3.84	10 ± 4.63

* 1a = hot water return line of Building 1, 1b = hot water return line of building 2, 1c = hot water return line of building 3.

## Supplementary Materials

The following are available online at https://www.mdpi.com/2076-0817/8/4/209/s1. Figure S1. Site map of MCH, Cotignola (RA), Italy. A representative view of MCH and a map of its three buildings: MCH picture (A) and MCH planimetry (B); Table S1. MCH structure and water outlet characteristics.

## Availability of Data Materials

The datasets generated and analyzed during the study are not publicly available due to ongoing further analysis on the data and protected by hospital privacy policies. The corresponding authors can response to any further questions about them.

## References

[1] Hoffman P., Friedman H., Bendinelli M. (2008). Legionella Pneumophila: Pathogenesis and Immunity.

[2] Cervero-Aragó S., Rodríguez-Martínez S., Puertas-Bennasar A., Araujo R.M. (2015). Effect of Common Drinking Water Disinfectants, Chlorine and Heat, on Free Legionella and Amoebae-Associated Legionella. PLoS ONE.

[3] Blanc D., Carrara P., Zanetti G., Francioli P., Blanc D. (2005). Water disinfection with ozone, copper and silver ions, and temperature increase to control Legionella: Seven years of experience in a university teaching hospital. J. Hosp. Infect..

[4] Canals O., Serrano-Suárez A., Salvadó H., Méndez J., Cervero-Aragó S., Ruiz de Porras V., Dellundè J., Araujo R. (2015). Effect of chlorine and temperature on free-living protozoa in operational man-made water systems (cooling towers and hot sanitary water systems) in Catalonia. Environ. Sci. Pollut. Res. Int..

[5] Ministerial Decree 15 December 1990. Information System of Infectious and Diffusive Diseases (G.U. n.6 of 8 January 1991. http://www.salute.gov.it/imgs/C17normativa1357allegato.pdf.

[6] Rota M.C., Caporali M.G., Bella A., Scaturro M., Giannitelli S., Ricci M.L. Rapporto Annuale Sulla Legionellosi in Italia Nel 2017. Notiziario Ist. Super Sanità n.9, Volume 31. September 2018. http://www.legionellaonline.it/notiziario%20legionellosi%20_9_2018.pdf.

[7] Italian Health Ministry Guidelines for Prevention and Control of Legionellosis. Approvate in Conferenza Stato-Regioni Seduta Del 7 Maggio 2015. http://www.salute.gov.it/imgs/C_17_pubblicazioni_2362_allegato.pdf.

[8] Montagna M.T., De Giglio O., Napoli C., Diella G., Rutigliano S., Agodi A., Auxilia F., Baldovin T., Bisetto F., Arnoldo L. (2018). Control and prevention measures for legionellosis in hospitals: A cross-sectional survey in Italy. Environ. Res..

[9] Kim B., Anderson J., Mueller S., Gaines W., Kendall A. (2002). Literature review—Efficacy of various disinfectants against Legionella in water systems. Water Res..

[10] Borella P., Bargellini A., Marchegiano P., Vecchi E., Marchesi I. (2016). Hospital-acquired Legionella infections: An update on the procedures for controlling environmental contamination. Ann. Ig..

[11] Farhat M., Moletta-Denat M., Frère J., Onillon S., Trouilhé M.C., Robine E. (2012). Effects of disinfection on Legionella spp., eukarya, and biofilms in a hot water system. Appl. Environ. Microbiol..

[12] Cristino S., Legnani P.P., Leoni E. (2012). Plan for the control of Legionella infections in long-term care facilities: Role of environmental monitoring. Int. J. Hyg. Environ. Health.

[13] Bédard E., Boppe I., Kouamé S., Martin P., Pinsonneault L., Valiquette L., Racine J., Prévost M. (2016). Combination of Heat Shock and Enhanced Thermal Regime to Control the Growth of a Persistent Legionella pneumophila Strain. Pathogens.

[14] White G.C. (1999). The Handbook of Chlorination and Alternative Disinfectants.

[15] Florentin A., Hautemanière A., Hartemann P. (2011). Health effects of disinfection by-products in chlorinated swimming pools. Int. J. Hyg. Environ. Health.

[16] Lin Y.E., Stout J.E., Yu V.L. (2011). Controlling Legionella in Hospital Drinking Water: An Evidence-Based Review of Disinfection Methods. Infect. Control Hosp. Epidemiol..

[17] WHO (2005). Trihalomethanes in Drinking-Water. http://www.who.int/water_sanitation_health/dwq/chemicals/THM200605.pdf.

[18] Richardson S.D., Plewa M.J., Wagner E.D., Schoeny R., DeMarini D.M. (2007). Occurrence, genotoxicity, and carcinogenicity of regulated and emerging disinfection by-products in drinking water: A review and roadmap for research. Mutat. Res..

[19] Smith E.M., Plewa M.J., Lindell C.L., Richardson S.D., Mitch W.A. (2010). Comparison of Byproduct Formation in Waters Treated with Chlorine and Iodine: Relevance to Point-of-Use Treatment. Environ. Sci. Technol..

[20] World Health Organization (2014). Water Safety in Distribution Systems. WHO Library Cataloguing-in-Publication Data. http://www.who.int/water_sanitation_health/publications/WSH-distribution_system-20141114.pdf.

[21] Schoenen D. (2002). Role of disinfection in suppressing the spread of pathogens with drinking water: Possibilities and limitations. Water Res..

[22] Linley E., Denyer S.P., McDonnell G., Simons C., Maillard J.-Y. (2012). Use of hydrogen peroxide as a biocide: New consideration of its mechanisms of biocidal action. J. Antimicrob. Chemother..

[23] Baldry M. (1983). The bactericidal, fungicidal and sporicidal properties of hydrogen peroxide and peracetic acid. J. Appl. Bacteriol..

[24] Martin N.L., Bass P., Liss S.N. (2015). Antibacterial Properties and Mechanism of Activity of a Novel Silver-Stabilized Hydrogen Peroxide. PLoS ONE.

[25] Ricci M., Dell’Eva I., Scaturro M., Baruchelli P., De Ponte G., Losardo M., Ottaviani M., Guizzardi F., Cianciotto N., Kwaik Y., Edelstein P., Fields B., Geary D., Harrison T., Joseph C., Ratcliff R., Stout J., Swanson M. (2006). Six-Month Experience of Silver-Hydrogen Peroxide Treatment for Legionella Control in Two Nursing Home Water Systems. Legionella. State of the Art 30 Years after Its Recognition.

[26] McDonnell G., Russell A.D. (2001). Antiseptics and Disinfectants: Activity, Action, and Resistance. Clin. Microbiol. Rev..

[27] Regulation (EU) 528/2012 of the European Parliament and of the Council of 22 May 2012 Concerning the Making Available on the Market and Use of Biocide Products. http://eur-lex.europa.eu/legal-content/EN/TXT/PDF/?uri=CELEX:32012R0528&from=IT.

[28] Juven B.J., Pierson M.D. (1996). Antibacterial Effects of Hydrogen Peroxide and Methods for Its Detection and Quantitation. J. Food Prot..

[29] Slawson R.M., Van Dyke M.I., Lee H., Trevors J.T. (1992). Germanium and silver resistance, accumulation, and toxicity in microorganisms. Plasmid.

[30] Loo S.-L., Fane A.G., Lim T.-T., Krantz W.B., Liang Y.-N., Liu X., Hu X. (2013). Superabsorbent Cryogels Decorated with Silver Nanoparticles as a Novel Water Technology for Point-of-Use Disinfection. Environ. Sci. Technol..

[31] Ehdaie B., Krause C., Smith J.A. (2014). Porous Ceramic Tablet Embedded with Silver Nanopatches for Low-Cost Point-of-Use Water Purification. Environ. Sci. Technol..

[32] Tugulea A.-M., Berube D., Giddings M., Lemieux F., Hnatiw J., Priem J., Avramescu M.-L. (2014). Nano-silver in drinking water and drinking water sources: Stability and influences on disinfection by-product formation. Environ. Sci. Pollut. Res..

[33] World Health Organization (2011). Guidelines for Drinking-Water Quality. http://apps.who.int/iris/bitstream/10665/44584/1/9789241548151_eng.pdf.

[34] Pedahzur R., Lev O., Fattal B., Shuval H.I. (1995). The interaction of silver ions and hydrogen peroxide in the inactivation of E. coli: A preliminary evaluation of a new long acting residual drinking water disinfectant. Water Sci. Technol..

[35] Pedahzur R. (1997). Silver and hydrogen peroxide as potential drinking water disinfectants: Their bactericidal effects and possible modes of action. Water Sci. Technol..

[36] Nabizadeh R., Samadi N., Sadeghpour Z., Beikzadeh M. (2008). Feasibility study of using complex of hydrogen peroxide and silver for disinfecting swimming pool water and its environment. Iran. J. Environ. Health.

[37] Absalan A., Ehrampoush M., Davoudi M., Vakili T., Ebrahimi A. (2012). Antibacterial effects of hydrogen peroxide and silver composition on selected pathogenic enterobacteriaceae. Int. J. Environ. Health Eng..

[38] Thomas V., Bouchez T., Nicolas V., Robert S., Loret J., Levi Y. (2004). Amoebae in domestic water systems: Resistance to disinfection treatments and implication in Legionella persistence. J. Appl. Microbiol..

[39] Berry D., Xi C., Raskin L. (2006). Microbial ecology of drinking water distribution systems. Curr. Opin. Biotechnol..

[40] Ditommaso S., Giacomuzzi M., Ricciardi E., Zotti C.M. (2016). Efficacy of a Low Dose of Hydrogen Peroxide (Peroxy Ag^+^) for Continuous Treatment of Dental Unit Water Lines: Challenge Test with Legionella pneumophila Serogroup 1 in a Simulated Dental Unit Waterline. Int. J. Environ. Res. Public Health.

[41] Shuval H., Shenman R., Yarom R. (2009). An Innovative Method for the Control of Legionella Infections in the Hospital Hot Water Systems with a Stabilized Hydrogen Peroxide-Silver Formulation. Int. J. Infect. Control.

[42] Casini B., Aquino F., Totaro M., Miccoli M., Galli I., Manfredini L., Giustarini C., Costa A.L., Tuvo B., Valentini P. (2017). Application of Hydrogen Peroxide as an Innovative Method of Treatment for Legionella Control in a Hospital Water Network. Pathogens.

[43] Rasamiravaka T., Labtani Q., Duez P., El Jaziri M. (2015). The Formation of Biofilms by Pseudomonas aeruginosa: A Review of the Natural and Synthetic Compounds Interfering with Control Mechanisms. BioMed Res. Int..

[44] Duda S., Baron J.L., Wagener M.M., Vidic R.D., Stout J.E. (2015). Lack of correlation between Legionella colonization and microbial population quantification using heterotrophic plate count and adenosine triphosphate bioluminescence measurement. Environ. Monit. Assess..

[45] Commission Regulation (EC) 1451/2007. http://eur-lex.europa.eu/legal-content/IT/TXT/PDF/?uri=CELEX:32007R1451&from=it.

[46] Decreto Legislativo 2 febbraio 2001, n. 31, Attuazione Della Direttiva 98/83/CE Relativa Alla Qualità Delle Acque Destinate al Consumo Umano (G.U. n. 52 Del 3 Marzo 2001—s.o.n. 41). http://www.gazzettaufficiale.it/eli/id/2001/03/03/001G0074/sg.

[47] Marchesi I., Ferranti G., Mansi A., Marcelloni A.M., Proietto A.R., Saini N., Borella P., Bargellini A. (2016). Control of Legionella Contamination and Risk of Corrosion in Hospital Water Networks following Various Disinfection Procedures. Appl. Environ. Microbiol..

[48] Mahapatra A., Padhi N., Mahapatra D., Bhatt M., Sahoo D., Jena S., Dash D., Chayani N. (2015). Study of Biofilm in Bacteria from Water Pipelines. J. Clin. Diagn. Res..

[49] Sabria M., Yu V.L. (2002). Hospital-acquired legionellosis: Solutions for a preventable infection. Lancet Infect. Dis..

[50] Douterelo I., Sharpe R., Boxall J. (2013). Influence of hydraulic regimes on bacterial community structure and composition in an experimental drinking water distribution system. Water Res..

[51] Liu S., Gunawan C., Barraud N., Rice S.A., Harry E.J., Amal R. (2016). Understanding, Monitoring, and Controlling Biofilm Growth in Drinking Water Distribution Systems. Environ. Sci. Technol..

[52] Rogers J., Dowsett A.B., Dennis P.J., Lee J.V., Keevil C.W. (1994). Influence of Plumbing Materials on Biofilm Formation and Growth of Legionella pneumophila in Potable Water Systems. Appl. Environ. Microbiol..

[53] Lehtola M.J., Laxander M., Miettinen I.T., Hirvonen A., Vartiainen T., Martikainen P.J. (2006). The effects of changing water flow velocity on the formation of biofilms and water quality in pilot distribution system consisting of copper or polyethylene pipes. Water Res..

[54] Yu J., Kim D., Lee T. (2010). Microbial diversity in biofilms on water distribution pipes of different materials. Water Sci. Technol..

[55] Rożej A., Cydzik-Kwiatkowska A., Kowalska B., Kowalski D. (2015). Structure and microbial diversity of biofilms on different pipe materials of a model drinking water distribution systems. World J. Microbiol. Biotechnol..

[56] Schwake D.O., Alum A., Abbaszadegan M. (2015). Impact of Environmental Factors on Legionella Populations in Drinking Water. Pathogens.

[57] Wingender J., Flemming H.-C. (2004). Contamination potential of drinking water distribution network biofilms. Water Sci. Technol..

[58] Fields B.S., Benson R.F., Besser R.E. (2002). Legionella and Legionnaires’ Disease: 25 Years of Investigation. Clin. Microbiol. Rev..

[59] Bartram J., Chartlier Y., Lee J.V., Pond K., Surman-Lee S. (2007). Legionella and the Prevention of Legionellosis.

[60] Brenner D.J., Steigerwalt A.G., Gorman G.W., Wilkinson H.W., Bibb W.F., Hackel M., Tyndall R.L., Campbell J., Feeley J.C., Thacker W.L. (1985). Ten new species of *Legionella*. Int. J. Syst. Evol. Microbiol..

[61] Casati S., Conza L., Bruin J., Gaia V. (2010). Compost facilities as a reservoir of Legionella pneumophila and other Legionella species. Clin. Microbiol. Infect..

[62] Duda S., Kandiah S., Stout J.E., Baron J.L., Yassin M., Fabrizio M., Ferrelli J., Hariri R., Wagener M.M., Goepfert J. (2014). Evaluation of a New Monochloramine Generation System for Controlling Legionella in Building Hot Water Systems. Infect. Control Hosp. Epidemiol..

[63] Husband P., Boxall J. (2011). Asset deterioration and discolouration in water distribution systems. Water Res..

[64] El-Chakhtoura J., Saikaly P.E., Van Loosdrecht M.C.M., Vrouwenvelder J.S. (2018). Impact of Distribution and Network Flushing on the Drinking Water Microbiome. Front. Microbiol..

[65] Council of the European Union (1998). European Council Directive 98/83/EC of 3 November 1998 on the Quality of Water Intended for Human Consumption. Off. J. Eur. Community.

[66] Imlay J.A. (2013). The molecular mechanisms and physiological consequences of oxidative stress: Lessons from a model bacterium. Nat. Rev. Genet..

[67] Vatansever F., De Melo W.C., Avci P., Vecchio D., Sadasivam M., Gupta A., Chandran R., Karimi M., Parizotto N.A., Yin R. (2013). Antimicrobial strategies centered around reactive oxygen species—Bactericidal antibiotics, photodynamic therapy, and beyond. FEMS Microbiol. Rev..

[68] Pedahzur R., Katzenelson D., Barnea N., Lev O., Shuval H., Fattal B., Ulitzur S. (2000). The efficacy of long-lasting residual drinking water disinfectants based on hydrogen peroxide and silver. Water Sci. Technol..

[69] ISO 11731:2017 Water Quality—Enumeration of Legionella. https://www.iso.org/standard/61782.html.

[70] Kimura S., Tateda K., Ishii Y., Horikawa M., Miyairi S., Gotoh N., Ishiguro M., Yamaguchi K. (2009). Pseudomonas aeruginosa Las quorum sensing autoinducer suppresses growth and biofilm production in Legionella species. Microbiology.

[71] (2006). UNI EN ISO 16266:2006 Water Quality—Detection and Enumeration of Pseudomonas Aeruginosa—Method by Membrane Filtration. https://www.iso.org/standard/39272.html.

[72] National Research Council (2006). Drinking Water Distribution Systems: Assessing and Reducing Risks.

[73] (2001). UNI EN ISO 6222:2001 Water Quality—Enumeration of Culturable Micro-Organisms—Colony Count by Inoculation in a Nutrient Agar Culture Medium. https://www.iso.org/standard/28960.html.

[74] Ratcliff R.M., Lanser J.A., Manning P.A., Heuzenroeder M.W. (1998). Sequence-Based Classification Scheme for the Genus Legionella Targeting the mip Gene. J. Clin. Microbiol..

[75] Mentasti M., Fry N.K., Afshar B., Palepou-Foxley C., Naik F.C., Harrison T.G., Fry N. (2012). Application of Legionella pneumophila-specific quantitative real-time PCR combined with direct amplification and sequence-based typing in the diagnosis and epidemiological investigation of Legionnaires’ disease. Eur. J. Clin. Microbiol. Infect. Dis..

[76] Fry N.K., Alexiou-Daniel S., Bangsborg J.M., Bernander S., Castellani Pastoris M., Etienne J., Forsblom B., Gaia V., Helbig J.H., Lindsay D. (1999). A multicenter evaluation of genotypic methods for the epidemiological typing of Legionella pneumophila serogroup 1: Results of a pan-European study. Clin. Microbiol. Infec..

[77] Fry N., Afshar B., Bellamy W., Underwood A.P., Ratcliff R., Harrison T.G. (2007). Identification of Legionella spp. by 19 European reference laboratories: Results of the European Working Group for Legionella Infections External Quality Assessment Scheme using DNA sequencing of the macrophage infectivity potentiator gene and dedicated online tools. Clin. Microbiol. Infect..

[78] APAT (2003). Agenzia per la Protezione Dell’ambiente e per i Servizi Tecnici. Manuali e Linee Guida 29/2003: Metodi Analitici per le Acque. http://www.isprambiente.gov.it/it/pubblicazioni/manuali-e-linee-guida/metodi-analitici-per-le-acque.

