# Supplementary material for: Advances in Legionella Control by a New Formulation of Hydrogen Peroxide and Silver Salts in a Hospital Hot Water Network
Source: Pathogens. 2019 Oct 29;8(4):209. doi: 10.3390/pathogens8040209 (PMC6963979; doi:10.3390/pathogens8040209)
Supplement: Supplementary file 1 [file pathogens-08-00209-s001.pdf]

SUPPLEMENTARY MATERIALS

Advances in Legionella control by a new formulation of hydrogen peroxide and silver salts in a hospital hot water network

Luna Girolamini[1], Ada Dormi[2], Tiziana Pellati[3], Paolo Somaroli[3], Davide Montanari[4], Andrea Costa[4], Francesca Savelli[5], Andrea Martelli[5], Antonella Grottola[6], Giulia Fregni Serpini[6], Sandra Cristino[1]*

[1] Department of Biological, Geological, and Environmental Sciences, BiGeA, University of Bologna, Bologna, Italy Affiliation; luna.girolamini2@unibo.it (LG); sandra.cristino@unibo.it (SC);

[2] Department of Medical and Surgical Science, DIMEC, University of Bologna, Bologna, Bologna Italy; ada.dormi@unibo.it (AD);

[3] GVM Care & Research, Lugo di Ravenna, Ravenna, Italy. tpellati@gvm-engineering.it (TP); psomaroli@gvm-engineering.it (PS);

[4] Eta-Beta S. r. l., Forlì, Forlì Cesena, Italy. andrea@etabetasrl.com (AC); manutenzione-mch@gvmnet.it (DM);

[5] Water Team S. r. l., Cesena, Forlì Cesena, Italy. francesca@waterteam.it (FS); andream@waterteam.it (AM);

[6] Regional Reference Laboratory for Clinical Diagnosis of Legionellosis, Unit of Microbiology and Virology, Modena University Hospital, Modena, Italy. grottola.antonella@aou.mo.it (AG); fregniserpini.giulia@aou.mo.it (GFS).

* Correspondence: sandra.cristino@unibo.it; Tel.: +39-0512094811 (S.C.)

Figure S1: Site map of MCH, Cotignola (RA), Italy. A representative view of MCH and a map of its three buildings: MCH picture (A) and MCH planimetry (B).

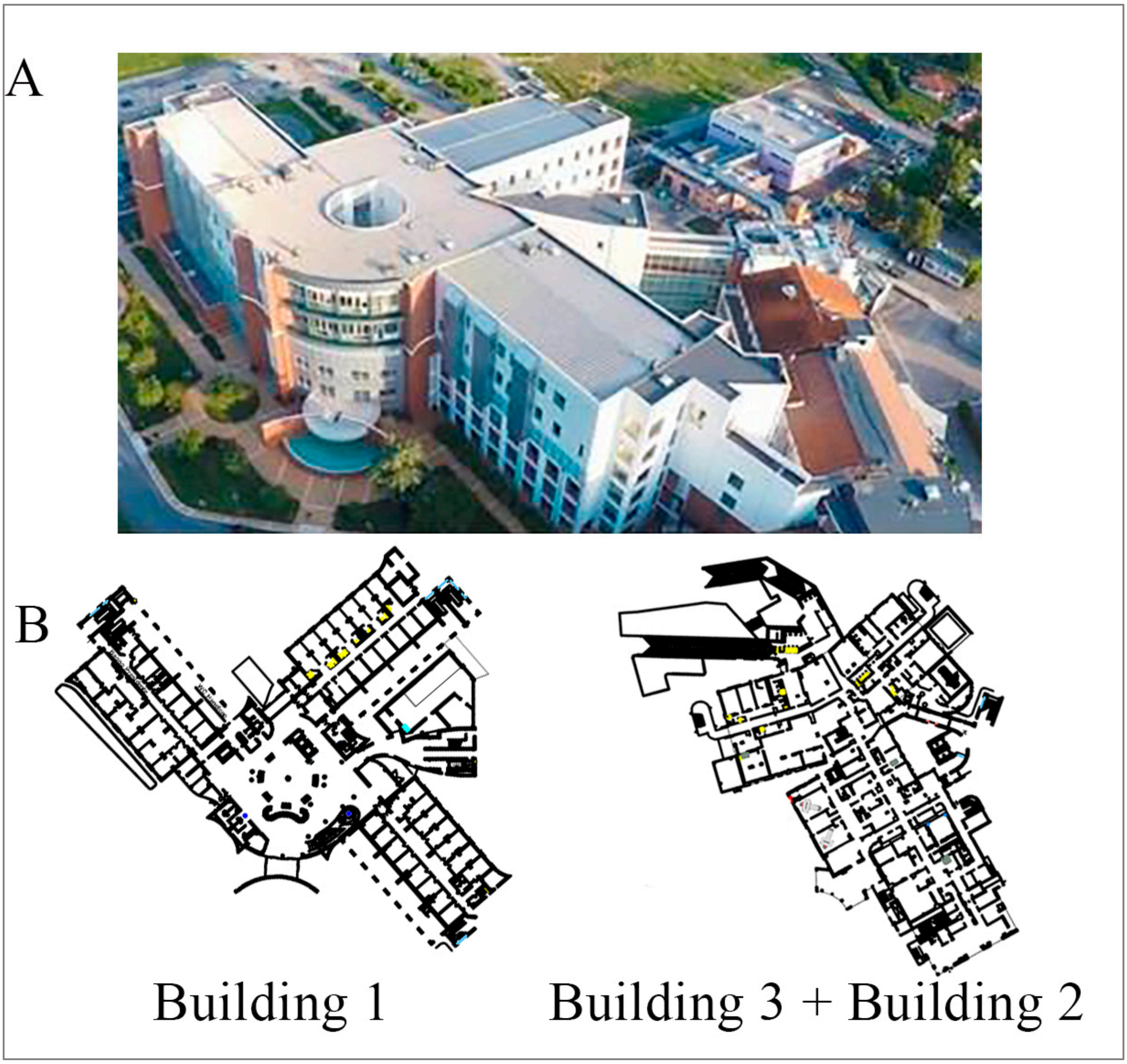

Table S1: MCH structure and water outlet characteristics

Building 1

Area (m^2)	Plumbing type	Year of construction/ year of works completion	Renovation time	Shock treatment	Floor	Water outlets	In-patient rooms	Sampling points	Water consumption (m^3)/ year
18539.93	Galvanized iron/ Polyvinylchloride (PVC) multilayer	2001/ 2015	2005 2010 2012 2013	Feb-Mar 2015 Jul-Aug 2015	Floor 0	50		4	1913
					Floor 1	25		4	
					Floor 2	119	27	5	
					Floor 3				
					Floor 4	73		4	
					Floor 5	36		4	
Total water outlets						303			
Total in-patient rooms							27		
Total sampling points								21	

Building 2

Area (m^2)	Plumbing type	Year of construction/ year of works completion	Renovation time	Shock treatment	Floor	Water outlets	In-patient rooms	Sampling points	Water consumption (m^3)/ year
8178.68	Galvanized iron	1973/ 1973	No works	No shock	Floor -1	4		1	3017
					Floor 0	65		5	
					Floor 1	92	25	5	
					Floor 2	104	26	5	
					Floor 3	66	18	3	
					Floor 4	5	1	2	
Total water outlets						336			
Total in-patient rooms							70		
Total sampling points								21	

Building 3

Area (m^2)	Plumbing type	Year of construction/ year of works completion	Renovation time	Shock treatment	Floor	Water outlets	In-patient rooms	Sampling points	Water consumption (m^3)/ year
1271.06	Galvanized iron/ Polyvinylchloride (PVC) multilayer	2001/ 2015	2001 2014 2015	No shock	Floor -1	7		2	589
					Floor 0	3		2	
					Floor 1	6		2	
					Floor 2	4		1	
					Floor 3	59	14	3	
					Floor 4	50	11	3	
Total water outlets						129			
Total in-patient rooms							25		
Total sampling points								13	
